# Enhancement of the Phase Transition Enthalpy of an Organic Phase Change Material Through the Use of Clinoptilolite

**DOI:** 10.3390/ma19091888

**Published:** 2026-05-03

**Authors:** Michał Musiał, Agnieszka Pękala, Lech Lichołai, Beata Mossety-Leszczak

**Affiliations:** 1 Faculty of Civil and Environmental Engineering and Architecture, Rzeszow University of Technology, Poznanska 2a, 35-029 Rzeszow, Poland; 2 Faculty of Chemistry, Rzeszow University of Technology, Warsaw Insurgents Avenue 6, 35-029 Rzeszow, Poland

**Keywords:** enthalpy variation, mineral composite, thermal energy storage, leakage resistance of liquid phase change materials, sustainable construction

## Abstract

**Highlights:**

- Prevention of phase change material leakage through the microstructure of clinoptilolite.
- The microstructure of clinoptilolite mitigates leakage of phase change materials.
- Increase in the phase change enthalpy of the composite.

**Abstract:**

The article presents a novel, energy-efficient composite of clinoptilolite and an organic phase change material (PCM), exhibiting a greater heat storage capacity than would be expected based solely on the PCM content within the composite. The study included a structural and textural analysis of clinoptilolite powder as a fine-grained material, with particular emphasis on its properties and compatibility with paraffin-based phase change materials. The second stage of the research involved determining changes in the enthalpy of melting and solidification of the composites, as well as evaluating their ability to retain the liquid phase and confirming the absence of chemical reactions between individual composite components. The obtained results demonstrated an increase in the enthalpy of the composite by approximately 14% and 44% relative to the expected values for PCM contents of 50% and 40%, respectively. Furthermore, the approximate content of paraffin-based PCM in the clinoptilolite composite at which no leakage occurs during the melting process was determined. This work represents a new approach to the integration of porous materials and phase change materials, enabling the formation of energetically favorable structures that significantly enhance the effective thermal storage capacity of PCM-based composites.

## 1. Introduction

Contemporary sustainable construction is founded on the implementation of sustainable development principles across economic, social, and environmental dimensions. A material and technological solution that simultaneously addresses all of these aspects is the storage of thermal energy derived from renewable sources. Enhancing the thermal energy storage capacity of buildings enables the effective accumulation of heat and cooling obtained from renewable sources, thereby improving thermal comfort while reducing the operational energy costs of buildings. This is typically achieved through the use of technologies, materials or dedicated thermal energy storage systems that incorporate substances characterised by high heat storage potential [1,2]. Thermal energy storage can be realised through the utilization of either sensible heat or latent heat of phase transition of specific materials [3]. Relying solely on sensible heat imposes significant limitations in terms of the amount of heat that can be stored, as well as considerable variations in the temperature range over which storage occurs. An alternative approach involves the use of the phase transition enthalpy of phase change materials (PCMs). In this case, heat storage occurs within a defined and narrow temperature range determined by the physicochemical properties of the material. At the same time, PCMs allow the storage of substantially larger amounts of heat, for example, 150–280 J/g [4,5,6], compared to sensible heat storage, such as in water (4.19 J/g·K), concrete, reinforced concrete, or natural stone (0.8–1.1 J/gK) [4,7]. An additional advantage of employing PCMs for thermal energy storage is that the process can proceed isothermally within the material structure for an extended period until the ambient temperature exceeds the phase-transition threshold. This characteristic allows for a significant reduction in heat losses during storage. However, the use of PCMs as thermal energy storage media is also associated with certain limitations. These include, among others, the relatively low thermal conductivity of organic PCMs [4,8], the slow distribution of stored heat, and the challenges related to maintaining the liquid phase of the PCM at the point of application [9,10]. Addressing these issues has been the subject of numerous scientific studies [11,12].

### 1.1. Limited Applicability of PCMs

As demonstrated in [13], the incorporation of microcapsules containing a C14-chain alkane as an additive to concrete, in a volumetric range of 2–8%, allowed for a reduction in peak temperatures of elements containing the composite. Furthermore, no deterioration in the workability of the fresh concrete mixture or in the mechanical properties after hardening was observed. In [14], the effect of adding n-eicosane as a phase change material to ceramic bricks was investigated in terms of reducing both the in heating and cooling energy demand for indoor spaces under various air conditioning systems. The most significant reduction in cooling demand of 31% was achieved in the absence of an air conditioning system and with eastern exposure of the composite.

Another example of liquid PCM application is described in [15], where natural cork granulate was combined with a cementitious matrix. The resulting composite was evaluated as a mortar with improved thermal storage capacity. The results demonstrated a partial mitigation of liquid PCM leakage. An example of using PCM in a free form is presented in [16], where propyl palmitate was added to organic foamed concrete. The study showed that vacuum impregnation and a PCM weight fraction below 35% prevented any PCM leakage. A significant limitation in the use of PCMs in microgranule form is the reduction in composite strength when the PCM content is excessive. Results presented in [17] demonstrated that the addition of 15 wt.% PCM microgranules to a cement-based mortar led to a decrease in compressive strength of up to 50%. To mitigate the negative impact of PCM on composite strength, silica fume and multi-walled carbon nanotubes were incorporated. This approach simultaneously increased the thermal conductivity of the composite and enhanced its compressive strength despite the presence of PCM. A similar trend of reduced compressive strength was observed in [18], where octadecane was applied directly together with biochar and cement or gypsum matrices. While the resulting composite exhibited satisfactory retention of PCM within its structure, its compressive strength was reduced by approximately 45%.

### 1.2. Properties of Microcapsules and Their Shells

A critical aspect of the applicability of PCMs in microcapsule form is the selection of an appropriate polymer for the capsule shell. In [19], the use of polyacrylonitrile (PAN) fibers was proposed, which enabled an increase in the tensile strength of the material containing PCM microcapsules by 15%.

### 1.3. Limitations in the Use of Inorganic PCMs Due to Congruency Phenomena

An example of inorganic PCM application in construction is described in [20], where hydrated barium hydroxide and expanded graphite were employed. The resulting composite did not exhibit congruent behavior and demonstrated a phase change enthalpy exceeding 250 J/g, along with an enhanced thermal conductivity, increasing from 0.63 W/m·K to 1.03 W/m·K.

### 1.4. Phase Change Materials in Thermal Management Applications

Beyond their application in building structures, PCMs are frequently employed as effective cooling systems for solar installations. In [21], analyses of the geometry of PCM-based heat exchangers in lithium ion battery packs were presented. The use of PCM combined with a pin-type heat exchanger resulted in a reduction in peak battery temperatures by 5 °C. A classical solution to the limited heat distribution of solid state PCMs involves the incorporation of high-thermal-conductivity additives, such as nanofibers, metal foams [22,23], aluminium and copper powders [24], or recycled conductive materials [25,26]. In [27], the addition of steel fibres at less than 0.1 wt.% delayed the achievement of the maximum temperature by 17–170%. For thermodynamic processes in PCM composites, in addition to the high thermal conductivity in the solid state, the geometry of the heat conductors is critical. As described in [28], when using copper foams with densities of 35, 80, and 95 pores per inch, the most effective pore fill height corresponded to approximately 1.3 times the foam thickness. Beyond providing a thermal conductor to the PCM, the form in which it is applied is also important. In [29], numerical simulations were conducted to evaluate the influence of the pore size of the metal foam on PCM heat distribution. The results indicated that increasing the dimensions of the copper foam enhanced the effective heat capacity of the PCM while simultaneously reducing the risk of supercooling. Another example of improving the heat distribution within organic PCMs is presented in [30], where biochar produced from the pyrolysis of hardwood, softwood and straw waste was employed. The study demonstrated that biochars with a highly porous structure, approximately 440 m^2^/g, effectively retain PCM within their matrix. Similarly, as reported in [31], the fabrication of a composite containing 15% expanded graphite within an epoxy resin matrix achieved thermal conductivity values of 9.03 W/m·K along the fibre direction and 5.58 W/m·K across the fibers. Furthermore, as shown in [32], expanded graphite can be used successfully to produce composites with inorganic phase change materials in the form of salt hydrates or their mixtures.

### 1.5. Ensuring the Integrity of Composites with Organic PCMs

An interesting application of phase-change materials is described in [33], where the thermal energy was captured through pressure-induced changes in PCM, which in turn affected the phase transition temperature. This approach enabled simultaneous thermal energy storage and its distribution against the temperature gradient. The integrity of the PCM was maintained as a result of the hydrophobic nature of the organic PCM used. In [34], a hybrid composite combining nonadecane PCM with expanded graphite and activated carbon was proposed. The resulting composite contained up to 50% PCM while maintaining its integrity in the liquid state and exhibited a phase change enthalpy of 173.11 J/g. A significant factor extending the applicability of PCMs beyond building applications is the development of flexible PCM composites, as reported in [35]. The combination of PCM with fibrous polymers produced a flexible mat with a thermal storage capacity of 87–90 J/g, representing a considerable limitation for high-capacity heat storage. Highly porous natural materials with the potential to retain PCM within their microstructure include minerals such as clinoptilolite.

**Figure 1:**
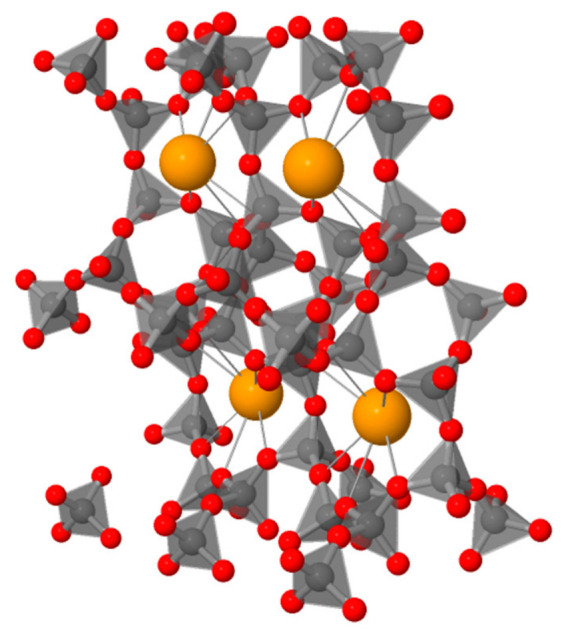
Schematic representation of the 3D microstructure of clinoptilolite [43].

Clinoptilolite, a high-silica zeolite mineral, is currently used in construction as a pozzolanic additive in cement and mortars [36,37,38,39,40,41]. It has been observed that the addition of zeolites to cement mixtures influences the heat of hydration and setting, as well as compressive strength, water resistance, and durability against corrosive agents. Studies have shown that samples with 10% zeolite addition exhibited a twofold increase in compressive strength compared to mortars without additive. The autoclaving process was particularly significant, enhancing the mechanical properties and demonstrating the potential for using this additive in aerated concretes [42]. Natural clinoptilolite and its modified forms can also serve as carriers for noble or transition metals. Such metal-loaded clinoptilolite catalysts can accelerate reactions that include isomerisation, alkylation, hydrogenation, and desulfurization. In its protonated (H^+^) form, clinoptilolite exhibits a high catalytic activity in alcohol dehydration reactions. Clinoptilolite belongs to the heulandite (HEU) group. Due to its crystal structure, it demonstrates strong adsorption capacity and ion-exchange properties, making it a versatile material applied across various industries as both an adsorbent and a catalyst. Clinoptilolite, a framework aluminosilicate zeolite, contains voids filled with large ions and water molecules that possess considerable mobility, enabling ion exchange and reversible dehydration. The fundamental building units of the zeolite framework are tetrahedra, whose centres are occupied by silicon or aluminium atoms, while the corners of the tetrahedra are occupied by oxygen atoms. Collectively, these tetrahedra form a continuous three-dimensional framework. Zeolite frameworks feature a variety of channels and cavities. Cavities generally adopt polyhedral shapes within which large free volumes exist, as illustrated in the microstructural architecture of clinoptilolite (Figure 1) [43]. Consequently, molecules can pass through the channel windows from one cavity to another, effectively moving between interconnected two-dimensional channel networks. The structural matrix of clinoptilolite, with the chemical composition (K_2_,Na_2_,Ca)_3_[Al_6_Si_30_O_72_]·~20H_2_O, is composed of AlO_4_ and SiO_4_ tetrahedra arranged in layers with a thickness of b/2 ≈ 0.9 nm. These layers are connected by oxygen atoms within the symmetry plane, forming a three-dimensional framework Figure 1. The distribution of silicon and aluminium in the tetrahedral positions is partially ordered. Water molecules occupy seven distinct positions within the structure of the clinoptilolite. Calcium, sodium, and potassium cations are coordinated by water molecules and framework oxygen atoms, while magnesium ions reside within octahedral water clusters. Owing to this unique internal structure, clinoptilolite exhibits pronounced hydrophilic properties.

Due to its unique properties and structure, clinoptilolite can serve as a medium that enhances the retention of liquid PCM within its microstructure in comparison to other conventional porous materials. In the scientific literature [44,45,46], numerous studies have reported the use of PCMs in conjunction with materials or substances exhibiting high thermal conductivity, such as copper, aluminium alloys, carbon nanotubes, graphene, and selected polymers. However, no studies that employ clinoptilolite for this purpose have been reported. Based on a comprehensive review of current knowledge on phase change material composites and the structures of natural and highly processed porous materials, a decision was made to develop an energetically advantageous composite based on paraffin and clinoptilolite. An additional objective of the study was to verify the potential for the stable retention of liquid PCM within the clinoptilolite microstructure over multiple phase change cycles.

## 2. Materials and Methods

### 2.1. Materials

- Phase change material RT21HC, characterized by a melting/freezing enthalpy of 190 J/g and a phase change temperature range of 19–23 °C (Rubiterm GmbH, Berlin, Germany).
- Clinoptilolite (powder with a median particle diameter of 50 μm, Nanga, Blękwit, Poland).

### 2.2. Apparatus and Experimental Methods

- The enthalpy of phase transition of the composite and the corresponding temperature range were determined using a DSC1 instrument (from Mettler Toledo, Greifensee, Switzerland), using STARe System software, Versions 19.00, (Mettler Toledo). The DSC traces were recorded at a heating and cooling rate of 5 °C/min under a nitrogen flow of 60 mL/min. The DSC instrument was calibrated using indium and zinc standards supplied by Mettler Toledo.
- Clinoptilolite chemical composition analysis was performed using a wavelength-dispersive X-ray fluorescence spectrometer, Ecublens, Switzerland, ARL Perform’X (WDXRF), Ecublens, Switzerland, equipped with OXSAS, Version 2.8.2 (Optical Emission Analytical Software) for data acquisition and analysis.
- The macroscopic examination of the composites was carried out using a stereoscopic microscope (Olympus SZX-7), Hamburg, Germany, with a Galilean optical system and distortion-free plan apochromatic objectives, coupled with a high-sensitivity sCMOS microscope camera (Moticam PRO-S5), Hong Kong, and software for image acquisition and measurement.
- Structural and textural characteristics, as well as surface elemental distribution, were investigated using a MIRA3 scanning electron microscope (SEM) (Tescan), Brno Czech Republic. Natural clinoptilolite samples were used for these analyses.

### 2.3. Experimental Procedure

**Figure 2:**
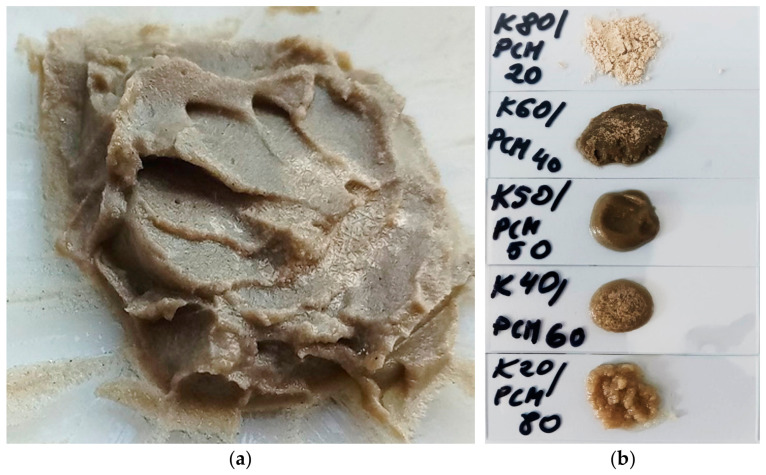
(**a**)—Composite sample composed of 50 wt% RT21HC PCM and 50 wt% clinoptilolite; (**b**)—Photograph of the Analyzed Composites.

The study was conducted empirically to evaluate the feasibility of combining powdered clinoptilolite with the organic hydrocarbon mixture RT21HC. In the first stage, four groups of test samples were prepared, differing in the weight ratio of PCM to clinoptilolite: 80:20, 60:40, 50:50, 40:60 and 20:80. Each sample had a total mass of 20 g. The individual components of the composite were initially combined at 20 °C, then heated to approximately 50 °C for 15 min. During this period, the components were mechanically mixed until a homogeneous consistency was achieved. Subsequently, the samples were cooled to 20 °C and reheated to 50 °C in cyclic repetitions of approximately 10 cycles. No changes in workability were observed during these repeated heating and cooling processes. Photographs of the PCM–clinoptilolite composite samples are presented in Figure 2.

After the initial sets of samples were prepared, those with PCM-to-clinoptilolite weight ratios of 80:20 and 20:80 were excluded from further analysis. The 80:20 samples were discarded due to the separation of liquid PCM from solid clinoptilolite particles, while the 20:80 samples were excluded because the clinoptilolite was not sufficiently saturated with PCM. The initially selected composite samples with PCM-to-clinoptilolite ratios of 40:60, as well as the newly prepared 50:50 samples, were subjected to microscopic observations during heating and cooling at a rate of 5 K/min. This enabled the monitoring of structural changes under thermal loading.

Subsequently, composite samples with weight ratios of 40:60 and 50:50 (PCM: clinoptilolite) were prepared. The components were initially combined at 20 °C and then heated to 50 °C, with the heating–cooling cycle repeated 10 times. The prepared samples were then subjected to differential scanning calorimetry (DSC), microscopic observations during thermal cycling, and scanning electron microscopy (SEM) analysis.

The next stage involved a detailed investigation of the chemical composition and structural and textural characteristics of the composites, focusing on optimal PCM retention and phase-change enthalpy. The sample preparation included coating with a thin layer of gold using a vacuum sputter coater. The imaging was performed at four magnifications, 2k×, 5k×, 20k×, and 50k×, with an accelerating voltage for the selected electrons in the range of 10–20 kV. Additionally, the SEM instrument was equipped with an EDS (energy-dispersive spectroscopy) detector, enabling elemental composition analysis.

## 3. Results

### 3.1. Structural and Textural Characteristics

**Figure 3:**
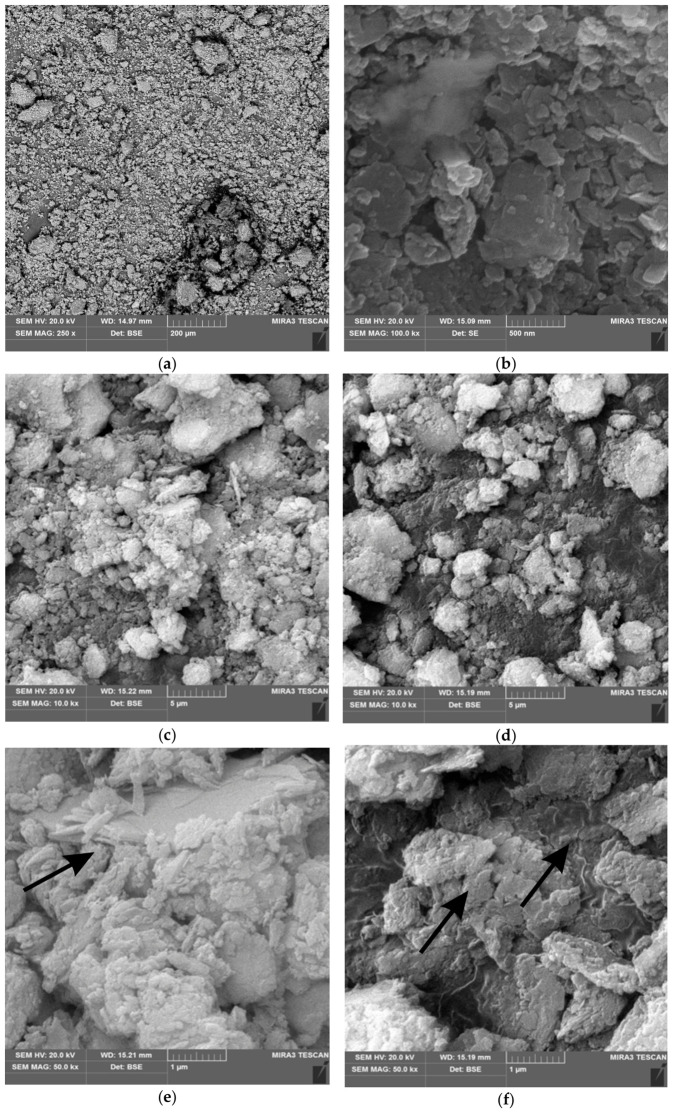
Morphology of the applied clinoptilolite, SEM images: (**a**) 200 µm, (**b**) 500 nm. At the 60/40 proportion, individual elongated, flat, and scattered clinoptilolite aggregates are visible (**c**,**e**). In a 50/50 ratio, visible clinoptilolite particles are covered with a filter and still bound to PCM (**d**,**f**).

The clinoptilolite used in this study is a natural high-silica zeolite in the form of a grey-green powder with 98% purity and particle sizes up to 50 μm. Surface morphology and structural imaging were recorded at magnifications of 200, 5, 1 µm, and 500 nm. SEM imaging revealed a plate-like morphology, with small, flat microcrystalline aggregates of clinoptilolite and a dispersed structure, as shown in Figure 3.

The results of scanning electron microscopy observations allow us to establish a certain relationship between the correlation mechanisms occurring in the newly formed composite. Clinoptilolite, as a powdery material, is clearly dispersed (Figure 3a,b). After creating mixtures of clinoptilolite and PCM in 50/50 and 60/40 proportions, SEM micrographs show a distinct modification of the composite surface. At the 60/40 proportion, individual elongated, flat, and scattered clinoptilolite aggregates are visible (Figure 3c,e). In the 50/50 proportion, the clinoptilolite material is clearly cemented with PCM. Flat clinoptilolite aggregates adsorb PCM, creating a rigid PCM composite structure through mechanical occlusion. At the microlevel, the mechanism of action of PCM and clinoptilolite is visible. Clinoptilolite particles are both covered with a filter and still bonded to the PCM, suggesting that interfacial bonds are formed (Figure 3d,f).

Scanning electron microscopy (SEM) examination illustrates the mechanism of action of the clinoptilolite/PCM mixture. SEM images clearly show a modification of the surface of the newly formed composite.

**Figure 4:**
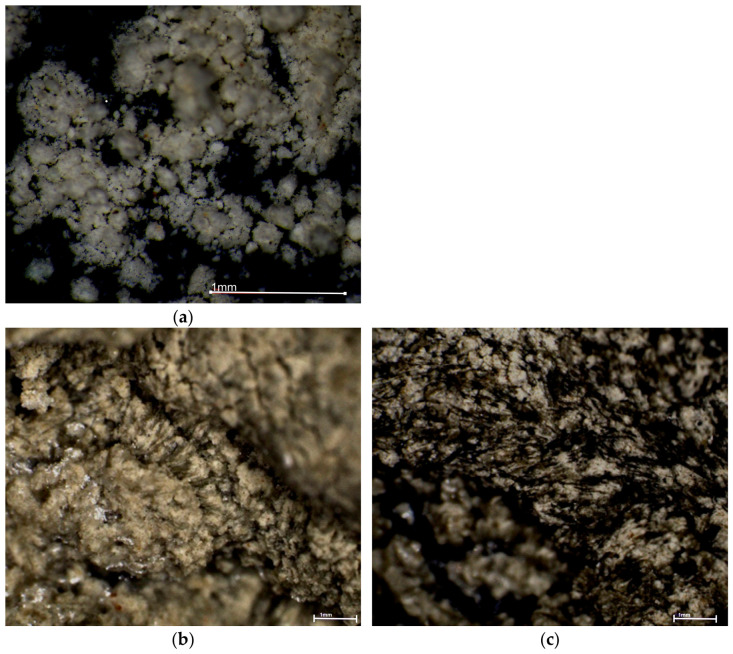
Structure of clinoptilolite (**a**), clinoptilolite–PCM composites (**b**) 50:50 ratio, (**c**) 60:40 ratio of clinoptilolite to PCM.

Stereoscopic microscopy was used to observe changes in the structural and textural features of the surfaces clinoptilolite and in the prepared clinoptilolite-based composites. In both the 50:50 and 60:40 weight ratio composites, feathery, spherulitic crystalline forms were evident (Figure 4). The PCM crystallized into fibrous, continuous structures within the clinoptilolite matrix, forming dense aggregates. In the 60:40 composite, the fibrous PCM structures were longer, creating a ‘scaffold’ for the clinoptilolite, resulting in a more compact surface with minimal free spaces.

### 3.2. Calorimetric Results of Composites

**Figure 5:**
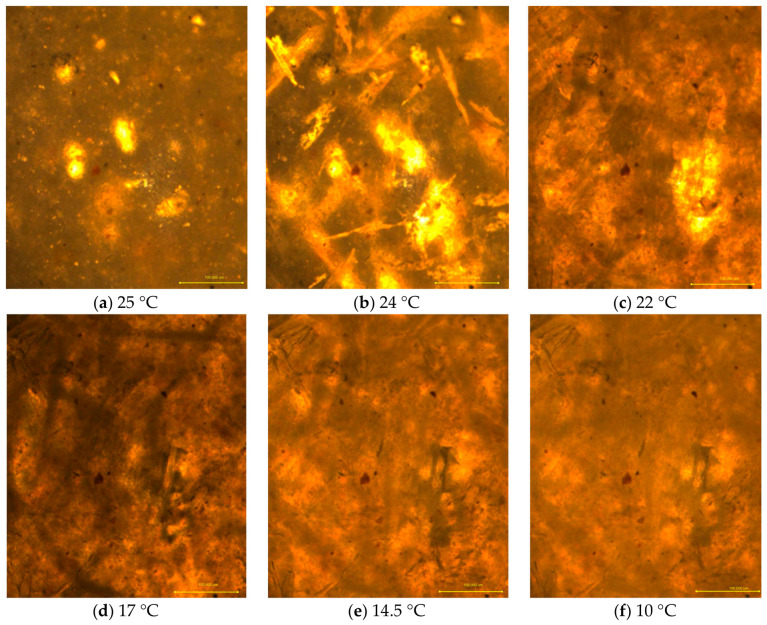
Micrographs of the composite containing 40% phase change material and 60% clinoptilolite during the cooling process.

Microscopic observations of the composite containing RT21HC and clinoptilolite during heating demonstrated the ability of clinoptilolite to retain PCM in its liquid state within the microstructure. Additionally, the formation of elongated, needle-shaped structures was observed in the composite after the PCM solidified. The process of needle-like structure formation during phase transitions of the PCM within the composite is illustrated in Figure 5.

As the temperature changes from 25 °C to 10 °C, a change in the state of matter occurs. At 24 °C, a distinct yellow fluorescence of PCM is visible in the micrograph (Figure 5b). The initial phase of PCM crystallisation is visible in the image (Figure 5) around the acicular clinoptilolite clusters. No changes are visible in the micrographs during subsequent cooling stages. The lack of significant differences in the DSC image in the micrographs at subsequent temperatures results from the fact that both components are already completely solid.

Calorimetric analysis of composites containing 40% PCM and 50% PCM revealed the presence of two distinct enthalpy peaks during the solidification of the composite: one at approximately 21–22 °C and a second at around 17–18 °C. Additionally, an increase in the temperature range of PCM melting was observed, expanding from ~4–5 °C to ~8–9 °C. The melting process occurred with a single dominant peak at approximately 27–28 °C.

**Figure 6:**
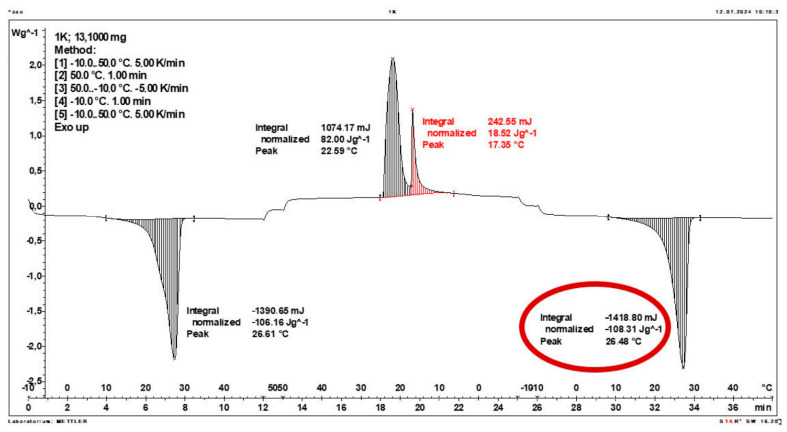
DSC calorimetric thermogram of the composite containing 50% phase change material and 50% clinoptilolite.

**Figure 7:**
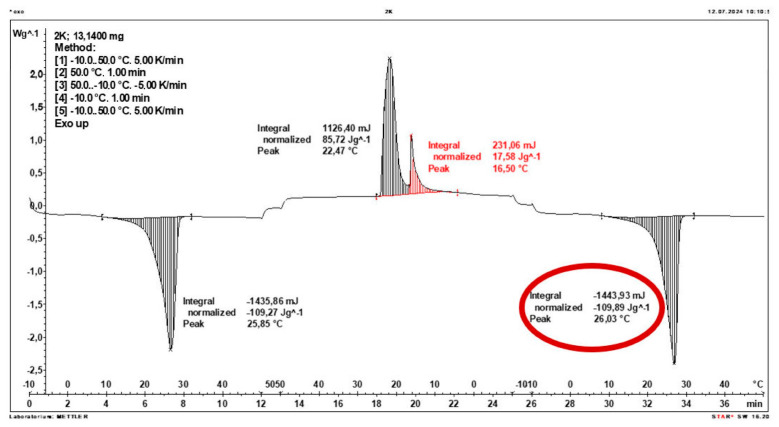
DSC calorimetric thermogram of the composite containing 40% phase change material and 60% clinoptilolite.

Furthermore, analysis of the enthalpy values recorded during DSC measurements demonstrated an increase in the actual enthalpy of the composite relative to the expected values based solely on the PCM content. This phenomenon was observed in samples containing 50% and 40% PCM by weight. For example, in Figure 6, where the PCM content is 50% (expected ΔH = 190 J/g × 50% = 95 J/g), the measured melting enthalpy was 108.31 J/g. A similar trend was observed in Figure 7, where the PCM content is 40% (expected ΔH = 190 J/g × 40% = 76 J/g), and the actual melting enthalpy was 109.89 J/g.

These results indicate an increase in the actual melting enthalpy of the composite relative to the expected values by 13.31 J/g for the 50% PCM samples and 33.89 J/g for the 40% PCM samples. This corresponds to an enhancement of 14.01% and 44.59%, respectively, compared to the anticipated enthalpy values.

Furthermore, the relative enthalpy values of the composites containing 50% and 40% PCM indicate that using a lower PCM content (40%) results in a more favourable heat storage potential compared to the composite with 50% PCM. Detailed calorimetric thermograms, along with the corresponding enthalpy values for composites containing 50% and 40% pure PCM, are presented in Figure 6 and Figure 7.

**Figure 8:**
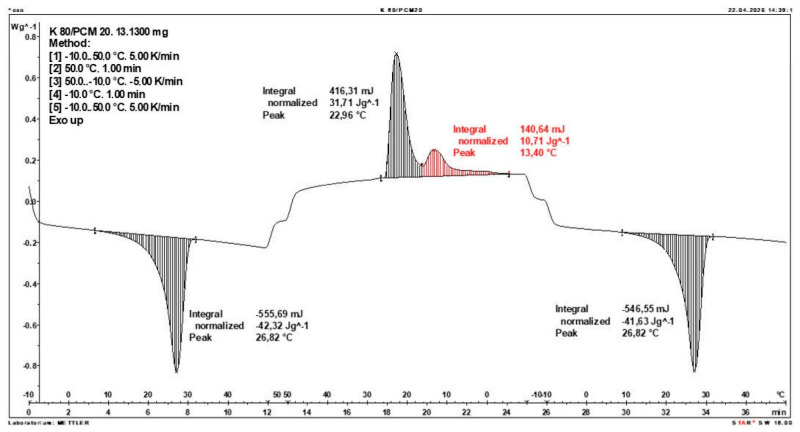
DSC Thermogram of the Composite Containing 20% PCM and 80% Clinoptilolite.

**Figure 9:**
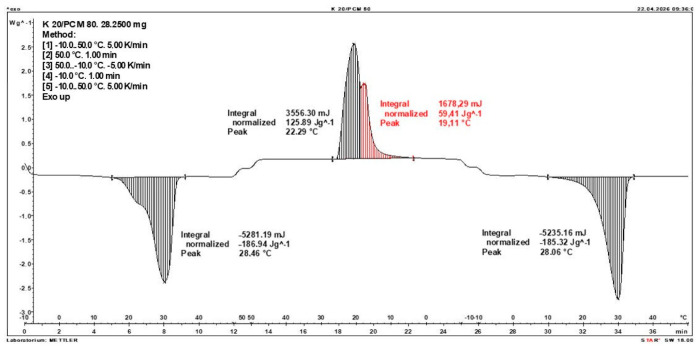
DSC Thermogram of the Composite Containing 80% PCM and 20% Clinoptilolite.

**Figure 10:**
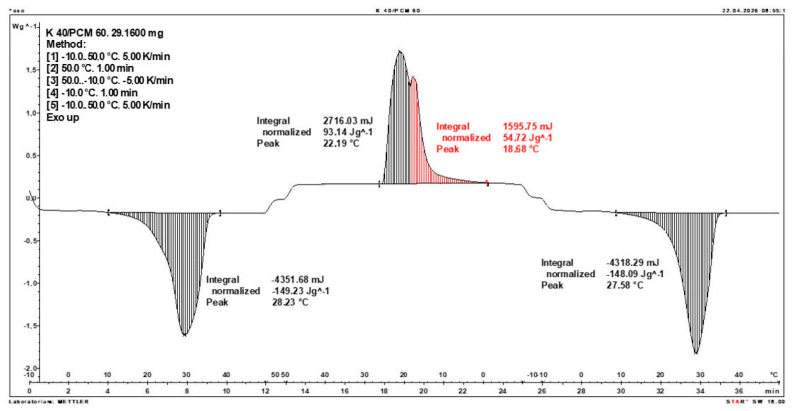
DSC Thermogram of the Composite Containing 60% PCM and 40% Clinoptilolite.

**Figure 11:**
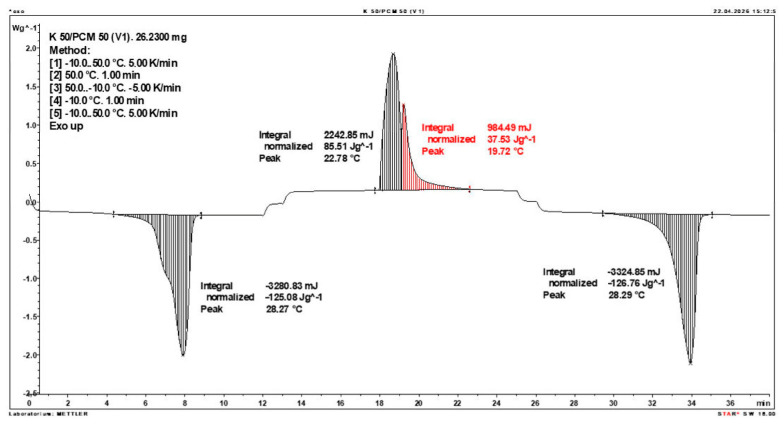
First Replicate of the DSC Thermogram of the Composite Containing 50% PCM and 50% Clinoptilolite.

**Figure 12:**
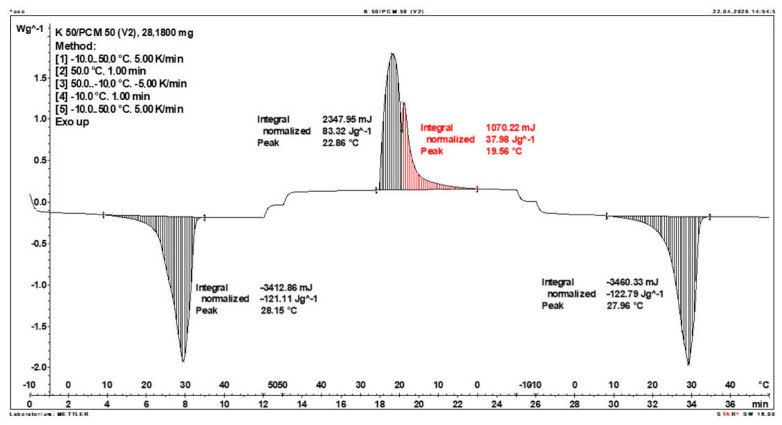
Second Replicate of the DSC Thermogram of the Composite Containing 50% PCM and 50% Clinoptilolite.

**Figure 13:**
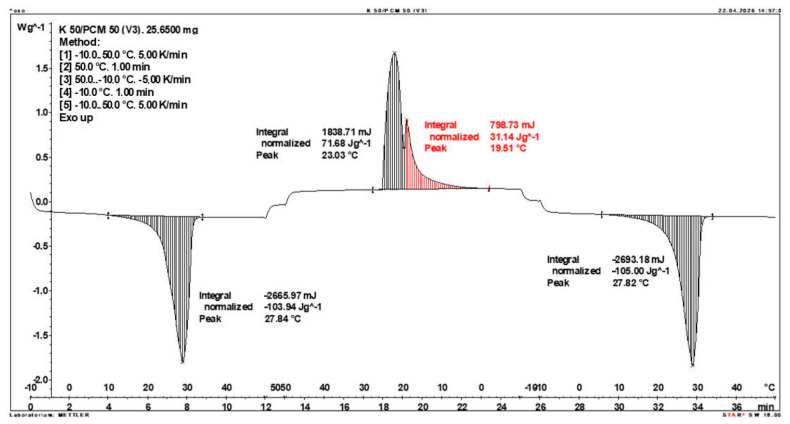
Third Replicate of the DSC Thermogram of the Composite Containing 50% PCM and 50% Clinoptilolite.

**Figure 14:**
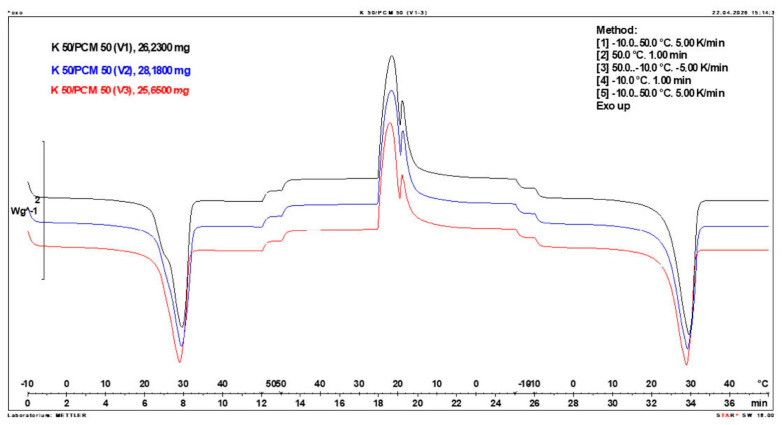
Comparison of Three DSC Calorimetric Curves Obtained from Separate Samples of the Composite Containing 50% PCM and 50% Clinoptilolite.

**Figure 15:**
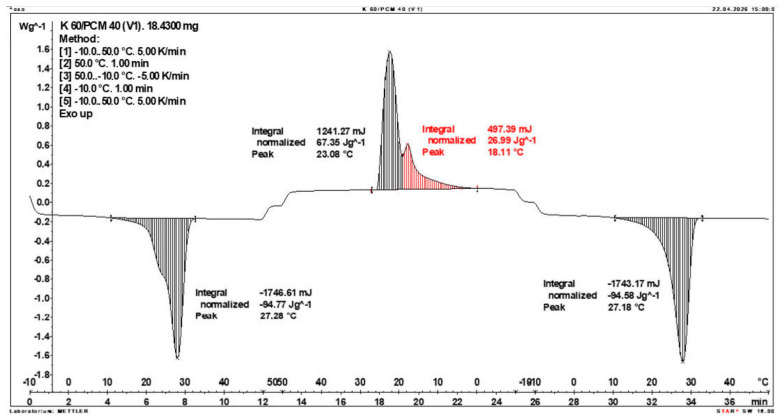
First Replicate of the DSC Thermogram of the Composite Containing 40% PCM and 60% Clinoptilolite.

**Figure 16:**
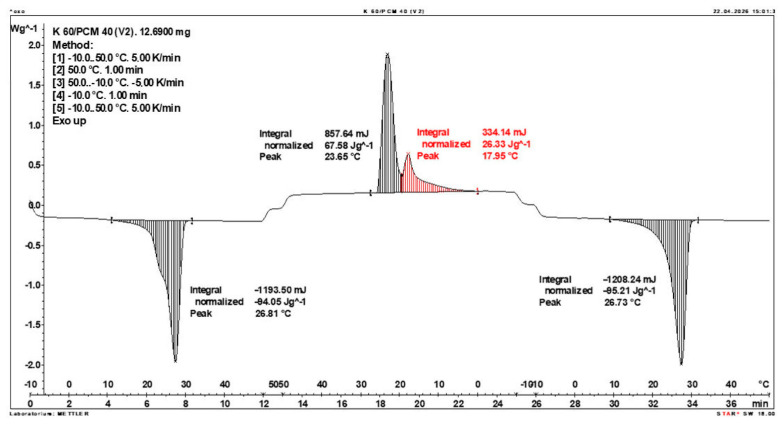
Second Replicate of the DSC Thermogram of the Composite Containing 40% PCM and 60% Clinoptilolite.

**Figure 17:**
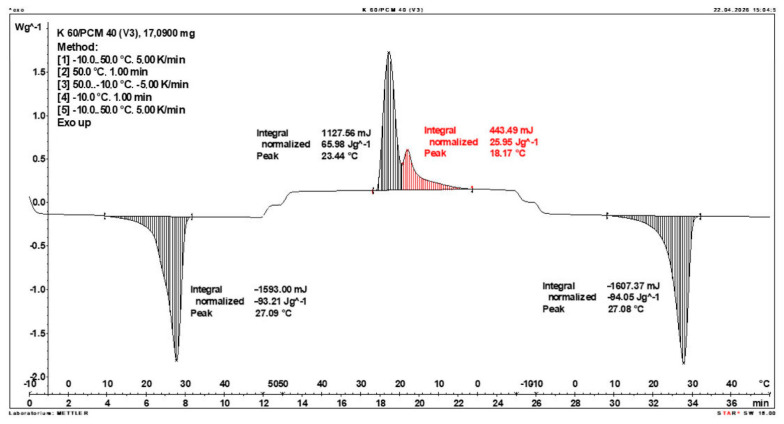
Third Replicate of the DSC Thermogram of the Composite Containing 40% PCM and 60% Clinoptilolite.

**Figure 18:**
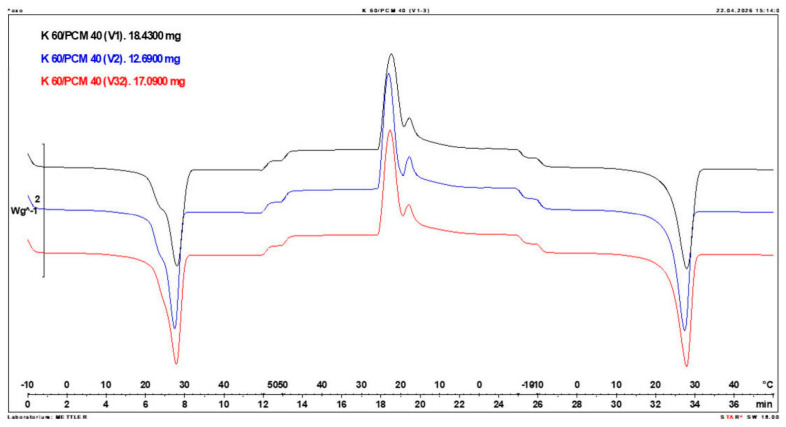
Comparison of Three DSC Calorimetric Curves Obtained from Separate Samples of the Composite Containing 40% PCM and 60% Clinoptilolite.

Additionally, to verify the obtained results, DSC measurements were repeated three times for samples containing 40% PCM and 50% PCM. Moreover, DSC analyses were also performed on composites that had been previously rejected due to their insufficient ability to bind PCM. The tests were conducted on separate, newly prepared composite samples. The obtained DSC results are presented in Figure 8, Figure 9, Figure 10, Figure 11, Figure 12, Figure 13, Figure 14, Figure 15, Figure 16, Figure 17 and Figure 18.

The observed increase in the phase transition enthalpy relative to the expected values in samples containing PCM and clinoptilolite results from the properties of clinoptilolite, which acts as a molecular sieve. As demonstrated in studies [47,48], clinoptilolite is capable of confining saturated aliphatic hydrocarbons within the channels of its structure, exhibiting a confinement effect. The pore and channel sizes of clinoptilolite enable the selective adsorption of alkanes with a specific carbon-chain length [49]. As a consequence of the selective uptake of individual chemical compounds from the RT21HC mixture, the relative proportions of the remaining components are altered. This, combined with the proton–acidic character of alkanes, may lead to changes in the enthalpy values of the mixture [50,51]. The presented results confirm the phenomenon of an increase in the phase transition enthalpy for each of the examined samples. However, the magnitude of this increase varies depending on the proportion of PCM and clinoptilolite, as well as on the resulting changes in the relative composition of the PCM mixture components that were not directly incorporated into the clinoptilolite microstructure.

### 3.3. Chemical Composition

**Figure 19:**
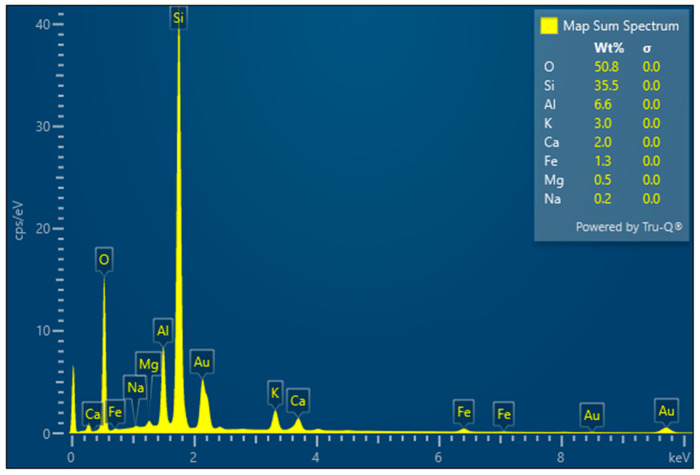
EDS spectrum showing the chemical composition with quantitative analysis of individual elements.

**Figure 20:**
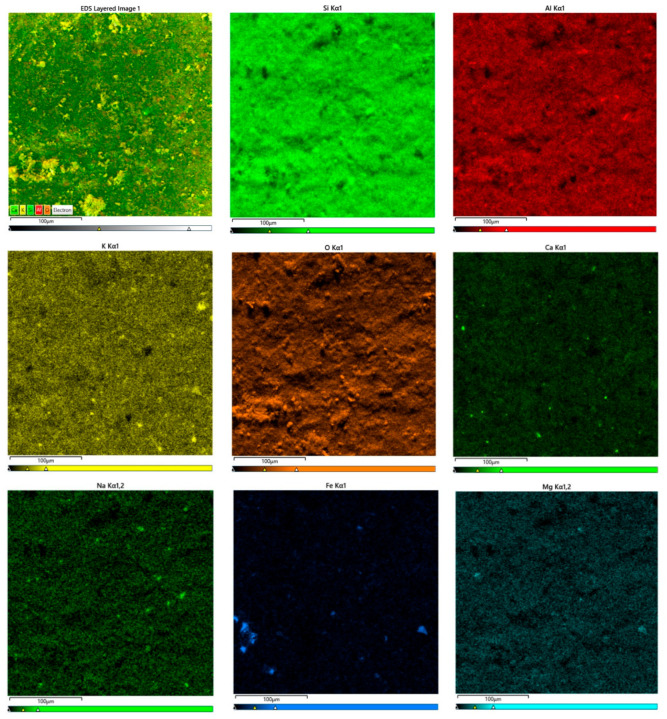
Elemental distribution on the surface of clinoptilolite, obtained by SEM/EDS.

Chemical analysis of the applied clinoptilolite revealed the following composition (by weight): SiO_2_—74.5%, Al_2_O_3_—13%, CaO—2.8%, K_2_O—3.1%, Fe_2_O_3_—2.7%, MgO—1.2%, Na_2_O—0.2%, MnO—0.08%, P_2_O_5_—0.03%. Elemental surface mapping using SEM/EDS confirmed its silico-aluminous character (Figure 19). The mapping images indicate a homogeneous coverage of Si and Al across the scanned surface. Minor localized accumulations of K, Ca, Na, and Fe were also observed, as shown in Figure 20. The identified chemical composition is typical for natural clinoptilolite-type zeolites. No additional chemical components that suggest contamination or toxicity were detected in the powdered material. Peaks corresponding to Au in the mapping spectra arise from the gold sputter coating applied for SEM analysis and do not reflect the native composition. All other peaks correspond to elements naturally present in the material structure. Both XRF and SEM/EDS analyses confirmed that silicon and aluminium are the dominant elements, validating the high-silica type of clinoptilolite. The EDS analysis results are consistent with the elemental composition. High-silica clinoptilolites also contain Na and K. In contrast, low-silica variants often contain Ba and Sr in addition to Ca; however, these elements were not detected in the analysed materials.

## 4. Discussion and Conclusions

The newly developed composite, owing to the unique microstructural architecture of clinoptilolite, enables not only efficient heat storage but also the incorporation of phase change material (PCM) within the large tetrahedral voids of the zeolite framework. Microscopic observations revealed the formation of fibrous, needle-like PCM structures that interconnect and bind the plate-like, fragmented clinoptilolite particles. Chemical analyses confirmed the high purity of the clinoptilolite, ensuring its suitability for safe integration with organic PCMs.

This property prevents the uncontrolled decomposition of aliphatic alkanes in the liquid state within the clinoptilolite structure. Consequently, the composite may be suitable for direct integration with other construction materials, which could represent a promising direction for future research. The study demonstrated an above-average retention capacity of liquid PCM in the form of saturated hydrocarbon mixtures within the clinoptilolite matrix. The maximum safe weight fraction of PCM relative to clinoptilolite, without risk of leakage, was determined to be 50%.

The most noteworthy result is the enhancement of the composite’s actual melting enthalpy: for composites containing PCM and clinoptilolite in 50:50 and 40:60 weight ratios, the enthalpy increased by 14.01% and 44.59%, respectively. Additionally, the composite with 40% PCM exhibited the highest absolute melting/crystallization enthalpy of 109.89 J/g, indicating its superior thermal storage efficiency.

These findings demonstrate that the clinoptilolite–PCM composite significantly expands the practical applications of free-form PCM in construction materials and highlights the potential to increase phase-change enthalpy through the formation of energetically favourable microstructures combining PCM and porous mineral matrices. The results contribute directly to the field of sustainable and energy-efficient construction, providing a pathway for the development of high-performance thermal energy storage composites that are safe, stable, and compatible with conventional building materials.

## Data Availability

The original contributions presented in this study are included in the article. Further inquiries can be directed to the corresponding author.

## Abbreviations

The following abbreviations are used in this manuscript:

PAN	Polyacrylonitrile
PCM	Phase-change materials
DSC	Differential Scanning Calorimetry
EDS	Energy Dispersive X-ray Spectroscopy
WDXRF	Wavelength Dispersive X-ray Fluorescence
SEM	Scanning Electron Microscopy
